# The effects of soy oil, poultry fat and tallow with fixed energy : protein ratio on broiler performance

**DOI:** 10.5194/aab-63-91-2020

**Published:** 2020-03-17

**Authors:** Nezih Okur

**Affiliations:** Department of Poultry Science, Bolu Abant Izzet Baysal University, Faculty of Agriculture and Natural Sciences, 14280, Bolu, Turkey

## Abstract

In this study, the effects of using soy oil (SO), poultry fat (PF) and tallow (T) in broiler feed at fixed energy : protein ratio on field and slaughter parameters were evaluated. The average live weight (ALW), feed conversion ratio (FCR), production efficiency factor (PEF) and mortality were investigated as field performance parameters; carcass weight (CW), carcass yield (CY), heart–liver weight (HLW), heart–liver yield (HLY), abdominal fat weight (AFW) and abdominal fat yield (AFY) were investigated as slaughter performance parameters. The experiment was performed in accordance with animal welfare legislation of Turkey and continued for 41 d. It was conducted with a total of 12 600 Ross 308 broiler chicks from Ross 308 strain middle-aged (36 weeks) broiler breeders. Ten different diets in which SO in starter; SO, PF and T in grower and single; or equal mixing of them (SO + PF, SO + T, PF + T) in finisher were used. When animal fat (PF and T) was used instead of SO, especially in grower feed, the field performance parameters improved except for mortality ( P<0.05 ). This situation was not seen in slaughter performance parameters except for CW, HLW and HLY ( P>0.05 ). However, it was found that sex affected slaughter performance parameters except for CY and AFW; higher CW and HLW and lower AFY and HLY were observed due to higher CW in male broilers ( P<0.05 ). In addition, the interactions between the type of the fat and sex were not found to be significant except for CW and CY ( P>0.05 ). At the end of the study, it was seen that if certain ratios are not exceeded, the use of animal fat instead of SO may be a good and economic alternative. Such an arrangement, which can be made depending on oil and fat prices, can reduce the feed cost, which is a more important result in terms of large integrations.

## 1 Introduction

In the poultry meat industry, which inevitably happens every day, production quantities increase by developing more efficient breeders, and increasing efforts are being made to reduce costs. While the efficiency parameters of breeders are one of the most important elements in poultry, growing, slaughtering and cutting-up of these efficient animals under optimum conditions in order to achieve these aims are also great importance.

The energy requirements of animals increase with increased production volumes and performance. Accordingly, the use of different oils and fats has become widespread due to increased prices of raw materials and vegetable oils. The use of fat in animal feeding has many advantages. Some of these advantages provide an intensive energy source to be used for increasing the energy ratios of the poultry diets, improving growth and feed conversion ratio (FCR) and reducing feed consumption due to less heat stress and higher heat resistance (Moura, 2003). However, there are some difficulties with using fat, such as balancing the feed energy, rancidity, additional equipment requirements, and low digestion especially in young chicks (Sell et al., 1986; Wiseman and Lesire, 1987; Aardsma et al., 2017).

Vegetable oils such as soy oil (SO) are preferred in poultry feeding while animal fats are used alternatively. Poultry fat (PF) and tallow (T) are the most important sources of animal fat. It should also be noted that when unsaturated fats such as SO are mixed with saturated fats such as PF and T, a synergistic effect is observed (Ketels and De Groote, 1989).

Used alone or mixed with other vegetable oils or animal fats in feeds, SO is one of the most important fat sources used in poultry diets after undergoing various processes such as filtration, hydration, degumming (Beauregard et al., 1996); digestibility of SO is higher than poultry fat (Aardsma et al., 2017) and T (Mahagna et al., 1988; Aardsma et al., 2017).

Poultry fat is obtained from slaughter by-products and non-edible organs by being processed in a rendering plant via a fat press. The ratio of unsaturated fatty acid in PF is reported to be higher than T (Aardsma et al., 2017). Poultry fat yield varies between 1.3 % and 1.6 % of average live weight (ALW) (Mano et al., 1999). In addition, when the diets with higher energy were used and slaughter age raised, higher ALW values were reached and a higher ratio of PF obtained from birds had higher ALW. Thus more PF was obtained from females than males (Baiao, 2005).

Tallow is obtained from sheep and bovines by methods similar to those used to obtain poultry fat. The ratios of saturated fatty acids such as palmitic acid and stearic acid are high in T (Aardsma et al., 2017). Tallow is especially effective when metabolic energy intake decreased and production is limited during heat stress conditions.

In the literature, when vegetable oils were used in poultry diets instead of animal fats, higher ME values were obtained. However, no difference was seen in the field performance parameters when different oil sources were used (Quart et al., 1992; Pesti et al., 2002). There can be several reasons for this. It was reported that when dietary energy : protein ratio and energy : amino acid ratio were held in equilibrium ratio, the type of oil did not significantly affect performance (Pinchasov and Nir, 1992; Leeson and Atteh, 1995; Pesti et al., 2002).

In practice, the use of fat in poultry diets is kept at a minimum level of 1 %. When cost is considered acceptable, an additional 1 % is included; where appropriate, the use of higher levels is recommended. In the USA, fat is generally added at 1 % to 3 % to starter diets, and the inclusion rates of fat are higher in finishing diets with rates increasing to 8 %–10 % (Firman, 2006).

Poultry fat is widely used in feed plant in the USA and many countries in the world, but prohibitions and specific rules on the use of PF in poultry feeds have caused these facilities to have issues with the EU and EU candidate countries (EC, 2004a, b, c). Due to the high amount of production, economic value, high nutrient content and high cost of alternative implementation, the by-product should be reintroduced into economy and production. Therefore, it is considered that legal regulations which provide continuous improvement in production, use and sales conditions according to current scientific developments should be continued, and the prohibitions put into effect should be reviewed again.

In the studies in which SO, T and combination of the two oils were used in female broilers, it was reported that the ALWs of the SO group were higher than the others (Scaife et al., 1994), while the abdominal fat weight (AFW) and abdominal fat yield (AFY) of T group were higher than the others (Sanz, 1999). In a study conducted on birds in which the control group was given a basal diet and the trial groups were given a diet containing SO and T for 8 weeks, higher ALW and lower FCR values were obtained in the SO group; when the fat rate increased, the difference between the groups reduced (Sell and Hodgson, 1962).

In a study which used diets containing SO, PF and combination of the two oils, no difference was observed between feed intakes and ALW (Dutra et al., 1991). In another study using SO, PF and combination of the two, better weight gain and feed intake was found in the SO group. However the FCR, mortality (Lara et al., 2003) and ALW (Moura, 2003) of the male broilers were found to be similar. In another study that used diets containing corn oil and PF, there was no significant difference in weight gain and FCR between the groups and values improved as oil levels increased (Valencia et al., 1993). No significant differences were found between carcass weights and the yields of the groups that were fed with SO and PF (Andreotti et al., 2001). In a study using SO, PF, T and their combinations, the ALWs of the SO group were higher (Aardsma et al., 2017).

In a study where SO, PF and T were given at the same ratio, it was found that the best fat and carcass quality were obtained in the SO group, the best FCR in the PF group and highest ALW values were in the T group (Azman et al., 2005). However, the slaughter performance parameters such as cold carcass weight (CW); the yield of breast, leg, and wing; and AFW did not change with fixed energy : protein ratio (Firman et al., 2008).

The aim of this study was to test whether PF or T can be used alone or in combination with SO to meet the metabolic energy needs, provide an economical feed product and increase the use of by-products.

## 2 Material and method

This study was performed in accordance with feed and animal welfare and legislation of Turkey. Efforts were made to prevent animal suffering. In order to better understand the materials and methods used in the experiment, segmentation was performed according to the basic materials and methods, and each section was designed within itself.

### 2.1 Test house

The growing phase of the experiment was carried out in a broiler test house of a company located in Bolu, Turkey. There were a total of 60 ground pens in the house, and each of the pens was 13.0 m ${}^{-2}$ (6.5 m × 2.0 m) and equipped with a pan feeder (MINIMAXline, Roxell NV, Belgium), a ceramic radiant heater (Rd 3 FA, SBM Int., France) and a nipple drinker (SPARKcup, Roxell NV, Belgium). In addition a digital scale connected to a central computer was set in each of the pens to monitor the live weight of the chickens throughout the experiment.

### 2.2 Feed production

For the experiment, the test feeds were formulated by adding SO, PF, T and their combinations at a fixed energy : protein ratio. PF was produced from processed poultry slaughterhouse by-products (blood, feathers, head, feet etc.) in a batch-cooker and fat pressed in a rendering unit of an integrated company located in Bolu, Turkey. SO and T were supplied from feedstuff suppliers.

Many issues were considered when designing the study. The first is the number of pens in the test house, which are the main constraints, and the number of replications that should be in the treatments. In the growing phase of the study, a test house with 60 pens and 210 broiler chicks in each was used. Although the minimum number of replications is four, the number of replications was six based our experience and as a precaution against unexpected problems during the growing phase of the study. If we had set the number of replications to 4, we could have run 15 different treatments, or we could increase the number of the treatments by dividing the pens again. However, we preferred to work with 210 birds in order not to decrease the number of birds in the replications, to avoid the problems in the stocking density, and to have a similar stocking density as run in field conditions.

One of the main objectives of the research is to study alternative fat sources to SO which will not cause problems or risk in terms of field performance, cutting yield and meat quality. Therefore, diets were formulated on an SO basis, and fat combinations were not preferred during the starter period. In this period, only one type of fat-containing diet formulas was preferred.

In terms of feed costs, being another issue, the research was based on that integrated poultry companies would want to prepare alternative feed formulas with their own oil resources, so PF was added to the study. Considering periods when the SO price is high, T alternatives are added as an option. Since PF is obtained within the integration, it is used as much as it can be used by integrations when the necessary conditions are formed. T, on the other hand, is usually supplied from different suppliers if it is suitable in terms of price and quality, and this is often not possible.

In this study, three fats (SO, PF and T) and one fat combination (SO + T) were used. In terms of feed, three different feeding phases (starter, grower and finisher/pre-slaughter) were used. In this case, the number of treatments can be 12 ( 4×3 ). Considering all possible combinations, the number of treatments to be studied would be much higher. For the limiting reasons described above, the number of treatments was 10 ( 60/6 ). In conclusion a total of 10 treatment groups that were designed based on their fat types and the trial design were included in the experiment (Table 1).

**Table 1:** Trial design and treatment groups in the experiment.

Feed type and fat source in feed			Group codes of treatments*
Starter	Grower	Finisher & pre-slaughter	
Soy oil*	Soy oil	Soy oil	SO + (SO)
Soy oil	Soy oil	Poultry fat	SO + (PF)
Soy oil	Soy oil	Tallow	SO + (T)
Soy oil	Soy oil	Soy oil + tallow	SO + (SO + T)
Soy oil	Poultry fat*	Soy oil	PF + (SO)
Soy oil	Poultry fat	Poultry fat	PF + (PF)
Soy oil	Poultry fat	Soy oil + tallow	PF + (SO + T)
Soy oil	Tallow*	Soy oil	T + (SO)
Soy oil	Tallow	Tallow	T + (T)
Soy oil	Tallow	Soy oil + tallow	T + (SO + T)

* SO: soy oil; PF: poultry fat; T: tallow.

The feeds were prepared with corn soya and manufactured in the feed mill of the same integrated company located in Bolu. SO, PF and T were added to the basal diet based on the trial design with fixed energy : protein ratios. Energy values of SO, PF and T based on diet formulations and analysis results made according to AOAC (2000) are given in Table 2.

**Table 2:** Energy values of soy oil, poultry fat and tallow based on diet formulations and analysis results.

	Soy oil	Poultry fat	Tallow
Moisture, %	0.80	0.97	0.95
Insoluble impurities, %	0.19	0.38	0.42
Free fatty acids, %	1.46	3.26	8.23
Peroxide value, meq kg ${}^{-1}$	0.85	1.42	3.95
Metabolic energy, kcal kg ${}^{-1}$	8700.00	8700.00	8700.00

The diets were formulated in accordance with international standards (NRC, 1994), and the recommendations of a grandparent company Ross (1.7–2.4 kg ALW) (Aviagen, 2014) were taken into consideration for the trial design. The feeds were manufactured in four phases: starter, grower, finisher and pre-slaughter. The starter feeds were produced in crumble form, while the other feeds were manufactured in pellet form (3.5 mm in diameter).

The raw material compositions of the prepared diets are given in Tables 3 and 4.

**Table 3:** Raw material compositions of the starter and grower feeds of the experiment.

Feed type	Starter ${}^{1}$	Grower ${}^{2}$		
Oil/fat source	Soy oil	Soy oil	Poultry fat	Tallow
Soy oil	10.00	18.00	0.00	0.00
Poultry fat	0.00	0.00	21.00	0.00
Tallow	0.00	0.00	0.00	21.00
Anticoccidial ${}^{3}$	0.50	0.50	0.50	0.50
Bonkalite	25.00	25.00	25.00	25.00
Broiler starter vitamin	1.00	0.00	0.00	0.00
Broiler chick vitamin	0.00	1.00	1.00	1.00
Choline chloride ${}^{4}$	0.36	0.34	0.34	0.34
Corn	472.81	513.64	508.72	508.72
DCP-18 ${}^{5}$	5.90	4.00	4.00	4.00
Fish meal	20.00	17.00	17.00	17.00
Full fat soybean meal	139.65	165.80	170.00	170.00
Gluten-60	12.00	11.00	11.00	11.00
Lysine ${}^{6}$	2.54	2.50	2.50	2.50
Marble powder	11.10	9.70	9.70	9.70
MDCP ${}^{5}$	6.50	4.40	4.40	4.40
Methionine-88 ${}^{7}$	1.50	1.00	1.00	1.00
Methionine-99 ${}^{8}$	2.02	1.84	1.84	1.84
Phytase enzyme ${}^{9}$	0.30	0.30	0.30	0.30
Poultry fat	0.00	0.00	21.00	0.00
Poultry offal meal	15.00	35.00	35.00	35.00
Poultry trace elements	1.00	1.00	1.00	1.00
Probiotic ${}^{10}$	1.00	0.00	0.00	0.00
Salt	2.00	2.00	2.00	2.00
Sodium bicarbonate	2.20	1.80	1.80	1.80
Soybean meal-48	217.12	128.68	126.40	126.40
Soy oil	10.00	18.00	0.00	0.00
Sunflower meal-36	15.00	15.00	15.00	15.00
Tallow	0.00	0.00	0.00	21.00
Wheat	35.00	40.00	40.00	40.00
Wheat enzyme ${}^{11}$	0.50	0.50	0.50	0.50
Total	1000.00	1000.00	1000.00	1000.00

${}^{1}$ Supplied per kg diet: Vit A 13000IU; Vit D3 5000IU; Vit E 100 mg; Vit B1 3 mg; Vit B2 8 mg; biotin 0.2 mg; Vit B6 5 mg; Vit B12 0.016 mg; Vit K3 4 mg; niacin 70 mg; folic acid 2 mg; Ca pantothenate 20 mg; Mn 120 mg; Zn 100 mg; Se 0.3 mg; Cu 16 mg; Fe 50 mg; I 2 mg and antioxidant 125 mg. ${}^{2}$ Supplied per kg diet: Vit A 11000IU; Vit D3 5000IU; Vit E 80 mg; Vit B1 2 mg; Vit B2 6 mg; biotin 0.2 mg; Vit B6 4 mg; Vit B12 0.016 mg; Vit K3 3 mg; niacin 70 mg; folic acid 1.75 mg; Ca pantothenate 20 mg; Mn 120 mg; Zn 100 mg; Se 0.3 mg; Cu 16 mg; Fe 50 mg; I 2 mg and antioxidant 125 mg. ${}^{3}$ Robenidine (% 6.6), anticoccidial (Lily Ilac., Istanbul, Turkey). ${}^{4}$ Liquid Choline Chloride, Choline-75 (Trouw Nutrition, Ankara, Turkey). ${}^{5}$ DCP-18 (di calcium phosphate, 18 % phosphate) and MDCP (mono di calcium phosphate, 21 % phosphate) (Rotem Turkey, Istanbul, Turkey). ${}^{6}$ Lysine-99, Lysine HCL 99 (Rotem Turkey, Istanbul, Turkey). ${}^{7}$ Dry Methionine, DL Methionine Feed Grade (Evonik Turkey, Istanbul, Turkey). ${}^{8}$ Liquid Methionine, Alimet (Novus Turkey, Istanbul, Turkey). ${}^{9}$ Phytase enzyme, Phyzyme (Nutriline Feed and Food Additives L.L.C., Istanbul, Turkey). ${}^{10}$ Probiotic, Diazyme W/S (Cuprem Inc., Nebraska, USA). ${}^{11}$ Wheat enzyme, Wheatzyme (Ekol, Inc., Turkey/Optivite International Ltd., UK).

**Table 4:** Raw material compositions of the finisher and pre-slaughter feeds used in the experiment.

Feed type	Finisher ${}^{1}$					Pre-slaughter ${}^{1}$				
Oil/fat source	SO*	PF*	T*	SO + PF	SO + T	SO*	PF*	T*	SO + PF	SO + T
Soy oil	22.00	0.00	0.00	12.00	12.00	22.00	0.00	0.00	12.00	12.00
Poultry fat	0.00	24.00	0.00	12.00	0.00	0.00	24.00	0.00	12.00	0.00
Tallow	0.00	0.00	24.00	0.00	12.00	0.00	0.00	24.00	0.00	12.00
Anticoccidial ${}^{2}$	0.50	0.50	0.50	0.50	0.50	0.00	0.00	0.00	0.00	0.00
Bonkalite	25.00	25.00	25.00	25.00	25.00	25.00	25.00	25.00	25.00	25.00
Broiler vitamin	1.00	1.00	1.00	1.00	1.00	1.00	1.00	1.00	1.00	1.00
Choline chloride ${}^{3}$	0.28	0.28	0.28	0.28	0.28	0.28	0.28	0.28	0.28	0.28
Corn	552.42	550.42	550.42	550.42	550.42	552.92	550.92	550.92	550.92	550.92
DCP-18 ${}^{4}$	3.10	3.10	3.10	3.10	3.10	3.10	3.10	3.10	3.10	3.10
Fish meal	12.00	12.00	12.00	12.00	12.00	12.00	12.00	12.00	12.00	12.00
Full fat soybean meal	191.78	191.78	191.78	191.78	191.78	191.78	191.78	191.78	191.78	191.78
Gluten-60	0.00	0.00	0.00	0.00	0.00	0.00	0.00	0.00	0.00	0.00
Lysine ${}^{5}$	0.56	0.56	0.56	0.56	0.56	0.56	0.56	0.56	0.56	0.56
MDCP ${}^{4}$	4.00	4.00	4.00	4.00	4.00	4.00	4.00	4.00	4.00	4.00
Marble powder	9.40	9.40	9.40	9.40	9.40	9.40	9.40	9.40	9.40	9.40
Methionine-88 ${}^{6}$	0.80	0.80	0.80	0.80	0.80	0.80	0.80	0.80	0.80	0.80
Methionine-99 ${}^{7}$	0.96	0.96	0.96	0.96	0.96	0.96	0.96	0.96	0.96	0.96
Phytase enzyme ${}^{8}$	0.30	0.30	0.30	0.30	0.30	0.30	0.30	0.30	0.30	0.30
Poultry offal meal	39.00	39.00	39.00	39.00	39.00	39.00	39.00	39.00	39.00	39.00
Poultry trace minerals	1.00	1.00	1.00	1.00	1.00	1.00	1.00	1.00	1.00	1.00
Salt	2.00	2.00	2.00	2.00	2.00	2.00	2.00	2.00	2.00	2.00
Sodium bi carbonate	1.80	1.80	1.80	1.80	1.80	1.80	1.80	1.80	1.80	1.80
Soybean meal-48	78.60	78.60	78.60	78.60	78.60	78.60	78.60	78.60	78.60	78.60
Sunflower meal-36	18.00	18.00	18.00	18.00	18.00	18.00	18.00	18.00	18.00	18.00
Wheat	35.00	35.00	35.00	35.00	35.00	35.00	35.00	35.00	35.00	35.00
Wheat enzyme ${}^{9}$	0.50	0.50	0.50	0.50	0.50	0.50	0.50	0.50	0.50	0.50
Total	1000.00	1000.00	1000.00	1000.00	1000.00	1000.00	1000.00	1000.00	1000.00	1000.00

* SO: soy oil; PF: poultry fat; T: tallow. ${}^{1}$ Supplied per kg diet: Vit A 11000IU; Vit D3 4000IU; Vit E 80 mg; Vit B1 2 mg; Vit B2 5 mg; biotin 0.05 mg; Vit B6 3 mg; Vit B12 0.012 mg; Vit K3 2 mg; niacin 40 mg; folic acid 1.5 mg; Ca pantothenate 20 mg; Mn 120 mg; Zn 100 mg; Se 0.3 mg; Cu 16 mg; Fe 50 mg; I 2 mg and antioxidant 125 mg. ${}^{2}$ Salinomycin, anticoccidial (Lily Ilac. Istanbul Turkey). ${}^{3}$ Liquid Choline Chloride, choline-75 (Trouw Nutrition Turkey, Ankara, Turkey). ${}^{4}$ DCP-18 (di calcium phosphate, 18 % phosphate) and MDCP (mono di calcium phosphate, 21 % phosphate) (Rotem Turkey, Istanbul, Turkey). ${}^{5}$ Lysine-99, lysine HCL 99 (Rotem Turkey, Istanbul, Turkey). ${}^{6}$ Dry Methionine, DL Methionine Feed Grade (Evonik Turkey, Istanbul, Turkey). ${}^{7}$ Liquid Methionine, Alimet (Novus Turkey, Istanbul, Turkey) ${}^{8}$ Phytase enzyme, Phyzyme (Nutriline Feed and Food Additives L.L.C., Istanbul, Turkey). ${}^{9}$ Wheat enzyme, Wheatzyme (Ekol Inc., Turkey/Optivite Int. Ltd., UK).

The prepared basal diets were analysed with AOAC (2000), and their specifications are given in Table 5. The basal diets formulations are based on SO. Other diets were formulated taking into account the energy value of PF and T; therefore they were not analysed separately.

**Table 5:** Feed specifications in the experiment (starter, grower, finisher and pre-slaughter).

	Starter	Grower	Finisher &
			pre-slaughter
Metabolic energy, kcal kg ${}^{-1}$	3025.00	3175.00	3250.00
Dry matter, %	88.81	88.92	88.93
Crude protein, %	23.10	20.50	19.00
Ether extract, %	7.18	8.98	9.75
Ash, %	6.48	5.84	5.49
Crude cellulose, %	3.83	3.73	3.68
Calcium, %	1.02	0.92	0.87
Phosphor, %	0.78	0.71	0.66
Available phosphor, %	0.51	0.46	0.43
Sodium, %	0.17	0.17	0.17
Chlorine, %	0.19	0.19	0.19
Methionine, %	0.54	0.53	0.43
Met + Cys, %	0.96	0.91	0.81
Lysine, %	1.40	1.27	1.07
Choline, %	1.81	1.62	1.41
Linoleic acid, %	3.54	4.44	4.77

### 2.3 Growing period

A total of 12 600 broiler chicks obtained from middle-aged (36 weeks) Ross 308 broiler breeders were used in the experiment.

The chicks used in the experiment were sexed, vaccinated and transferred to the test house. They were then weighed using a scale (EC-130, Bizerba SE/Co. KG, Germany) and randomly distributed to the 60 pens as 210 chicks per pen (stocking density was 16.15 chicks m ${}^{-2}$ ) with each subgroup consisting of 50 % male +50 % female. A total of six replications were used per treatment during the rearing period.

The broilers were fed in four phases: starter, grower, finisher and pre-slaughter on 0–11th, 12–23th, 24–36th and 37–41th days of growing, respectively. Feed and water were given ad libitum during the experiment.

The ALWs of the chicks were determined for each subgroup at the end of the experiment before loading for transport. The feed consumption levels of the subgroups were determined by weighing the feed in the feeders at the beginning and end of the rearing period. The FCR values were calculated following Eq. (1) (Aviagen, 2018):

1 $$\mathrm{FCR}=\frac{\text{total feed consumed, kg }}{\text{total live weight, kg}}.$$

The number of chicks that died on a daily basis in the subgroups was recorded, and the liveability and mortality rates were calculated for the trial period. Production efficiency factor (PEF) values were calculated by using liveability, live weight, age and FCR following Eq. (2) (Aviagen, 2018):

2 $$\mathrm{PEF}=\frac{\mathrm{liveability},\;\%\times \mathrm{live}\;\mathrm{weight}\;\mathrm{kg}}{\mathrm{age},\;\mathrm{day}\times \mathrm{FCR}}.$$

### 2.4 Slaughtering and cut-up process

At the end of the experiment, a total of 12 broilers including 6 males and 6 females close to the ALW values of their group were manually slaughtered, defeathered and eviscerated. The warm CWs were measured for each treatment group by a scale (EC-130, Bizerba SE/Co. KG, Germany). Then heart–liver weights (HLWs) and abdominal fat weights (AFW) were measured during this process; carcass yield (CY), heart–liver yield (HLY) and abdominal fat yield (AFY) were calculated individually for the same carcass samples.

### 2.5 Statistical analysis

The statistical analyses were performed by IBM SPSS Statistics 22 (SPSS, 2013). The Shapiro–Wilk test was used to confirm the normal distribution of the data. After this process, an analysis of variance (ANOVA) was conducted for the experiment using the GLM procedure of SPSS appropriate for one-way and two-way designs. The one-way ANOVA designs for field performance parameters and two-way ANOVA designs for slaughter performance parameters were used. One-way ANOVA model used was following Eq. (3):

3 $$Y_{ij}=\mu +F_{i}+e_{ij},$$

where $Y_{ij}$ is the dependent variable, μ is the overall mean, $F_{i}$ is the effect of fat sources ( ${}_{i}=$ SO + (SO), SO + (PF), SO + (T), SO + (SO + T), PF + (SO), PF + (PF), PF + (SO + T), T + (SO), T + (T) or T + (SO + T) in the experiment) and $e_{ij}$ is the random error term.

Two-way ANOVA model used follows Eq. (4):

4 $$Y_{ijk}=\mu +F_{i}+S_{j}+(\mathrm{FS}{)}_{ij}+e_{ijk},$$

where $Y_{ij}$ is the dependent variable, μ is the overall mean, $F_{i}$ is the effect of fat sources ( ${}_{i}=$ SO + (SO), SO + (PF), SO + (T), SO + (SO + T), PF + (SO), PF + (PF), PF + (SO + T), T + (SO), T + (T) or T + (SO + T) in the experiment), $S_{j}$ is the effect of sex ( j= female and male in the experiment and), (FS) ${}_{ij}$ is the interaction between fat and sex effect, and $e_{ijk}$ is the random error term.

Tukey's test was used to analyse the differences in the investigated parameters. P values less than 0.05 were considered as statistically significant. All the data were given as means ± standard error of the means (M ± SEM).

## 3 Results and discussion

In the present study, the effects of SO, PF and T used in broiler chickens at different levels on broiler field and slaughter performance parameters were investigated. ALW, FCR, PEF and mortality as field performance parameters and CW, CY, AFW, AFY, HLW and HLY as slaughter performance parameters were determined. The evaluation of the data obtained as a result of the research was conducted separately and is shown in the Tables 6 and 7.

**Table 6:** The effects of using soy oil, poultry fat and tallow in feeds at fixed energy : protein ratio on broilers' field performance parameters.

	ALW ${}^{1}$ (g)	FCR ${}^{1}$	PEF ${}^{1}$	Mortality (%)
Fat/oil source in feeds ${}^{2}$				
Grower + (finisher & pre-slaughter)				
SO + (SO)	2.323±22${}^{\text{bc}}$	1.843±0.010	296.72±1.92	3.46±0.93
SO + (PF)	2.346±31	1.819±0.014	300.08±6.59	4.64±1.02
SO + (T)	2.351±28	1.821±0.016	302.95±5.58	3.81±1.64
SO + (SO + T)	2.281±51${}^{\text{c}}$	1.858±0.035${}^{\text{a}}$	289.79±14.53${}^{\text{b}}$	3.56±1.23
PF + (SO)	2.391±28${}^{\text{ab}}$	1.805±0.007${}^{\text{b}}$	304.83±2.65	5.64±0.75
PF + (PF)	2.369±12${}^{\text{ab}}$	1.795±0.014${}^{\text{b}}$	312.67±3.53${}^{\text{a}}$	2.86±0.34${}^{\text{b}}$
PF + (SO + T)	2.360±23	1.822±0.004	300.76±2.54	4.78±0.71
T + (SO)	2.329±6	1.833±0.008	298.48±1.33	3.72±0.40
T + (T)	2.401±23${}^{\text{ab}}$	1.828±0.010	301.54±4.02	5.88±0.70
T + (SO + T)	2.412±6${}^{\text{a}}$	1.839±0.012	300.39±4.67	6.14±1.17${}^{\text{a}}$
p values	0.043	0.034	0.011	0.035

${}^{1}$ ALW: average live weight; FCR: feed conversion ratio; PEF: production efficiency factor. ${}^{2}$ SO: soy oil; PF: poultry fat; T: tallow. ${}^{\text{abc}}$ The difference between the averages indicated by different letters in the same column are statistically significant ( p<0.05 ).

**Table 7:** The effects of using soy oil, poultry fat and tallow in feeds at fixed energy : protein ratio on broilers' slaughter performance parameters.

		CW ${}^{1}$	CY ${}^{1}$	AFW ${}^{1}$	AFY ${}^{1}$	HLW ${}^{1}$	HLY ${}^{1}$
		g	%	g	%	g	%
Fat/oil source in feeds ${}^{2}$							
Grower + (finisher & pre-slaughter)							
SO + (SO)		1647 ± 35	67.56 ± 0.45	42 ± 2	2.49 ± 0.10	52 ± 2 ${}^{\text{b}}$	3.20 ± 0.08
SO + (PF)		1649 ± 34	67.53 ± 0.54	36 ± 3	2.23 ± 0.14	58 ± 2	3.56 ± 0.09 ${}^{\text{a}}$
SO + (T)		1657 ± 26	68.30 ± 0.32	43 ± 3	2.58 ± 0.23	52 ± 1 ${}^{\text{b}}$	3.10 ± 0.07 ${}^{\text{b}}$
SO + (SO + T)		1663 ± 23	68.41 ± 0.37	35 ± 2	2.10 ± 0.15	54 ± 1	3.27 ± 0.11
PF + (SO)		1665 ± 31	68.06 ± 0.30	37 ± 2	2.26 ± 0.17	56 ± 2	3.38 ± 0.11
PF + (PF)		1657 ± 32	67.72 ± 0.15	37 ± 2	2.25 ± 0.14	57 ± 2	3.45 ± 0.11
PF + (SO + T)		1700 ± 24 ${}^{\text{a}}$	68.56 ± 0.52	40 ± 2	2.34 ± 0.15	55 ± 1	3.23 ± 0.07
T + (SO)		1652 ± 34	67.41 ± 0.31	39 ± 2	2.38 ± 0.09	54 ± 1	3.37 ± 0.06
T + (T)		1682 ± 25 ${}^{\text{ab}}$	67.40 ± 0.34	39 ± 2	2.35 ± 0.15	60 ± 1 ${}^{\text{a}}$	3.57 ± 0.08 ${}^{\text{a}}$
T + (SO + T)		1627 ± 19 ${}^{\text{c}}$	67.97 ± 0.33	36 ± 3	2.20 ± 0.16	54 ± 1	3.28 ± 0.08
Sex	Female	1575 ± 6 ${}^{\text{b}}$	67.91 ± 0.18	39 ± 1	2.47 ± 0.07 ${}^{\text{a}}$	54 ± 1 ${}^{\text{b}}$	3.46 ± 0.04 ${}^{\text{a}}$
	Male	1744 ± 7 ${}^{\text{a}}$	67.88 ± 0.17	38 ± 1	2.16 ± 0.06 ${}^{\text{b}}$	57 ± 1 ${}^{\text{a}}$	3.26 ± 0.04 ${}^{\text{b}}$
Interaction, fat type × sex							
SO + (SO)	Female	1544 ± 25 ${}^{\text{d}}$	67.12 ± 0.68	42 ± 2	2.65 ± 0.12	50 ± 3	3.34 ± 0.14
SO + (PF)	Female	1544 ± 14 ${}^{\text{d}}$	66.66 ± 0.61	30 ± 2	1.96 ± 0.14	54 ± 1	3.53 ± 0.09
SO + (T)	Female	1578 ± 6 ${}^{\text{d}}$	68.16 ± 0.46	48 ± 5	3.03 ± 0.31	50 ± 2	3.17 ± 0.13
SO + (SO + T)	Female	1604 ± 24 ${}^{\text{cd}}$	68.87 ± 0.46	37 ± 2	2.32 ± 0.21	55 ± 2	3.48 ± 0.16
PF + (SO)	Female	1569 ± 12 ${}^{\text{d}}$	67.86 ± 0.57	41 ± 3	2.59 ± 0.20	56 ± 3	3.68 ± 0.20
PF + (PF)	Female	1555 ± 12 ${}^{\text{d}}$	67.63 ± 0.19	38 ± 3	2.47 ± 0.23	54 ± 3	3.47 ± 0.14
PF + (SO + T)	Female	1627 ± 14 ${}^{\text{cd}}$	69.23 ± 0.62 ${}^{\text{a}}$	41 ± 4	2.50 ± 0.23	56 ± 2	3.46 ± 0.09
T + (SO)	Female	1556 ± 11 ${}^{\text{d}}$	67.11 ± 0.46	37 ± 2	2.41 ± 0.08	53 ± 2	3.44 ± 0.09
T + (T)	Female	1602 ± 12 ${}^{\text{cd}}$	68.27 ± 0.41	39 ± 4	2.44 ± 0.27	60 ± 1	3.73 ± 0.07
T + (SO + T)	Female	1574 ± 9 ${}^{\text{d}}$	68.14 ± 0.50	36 ± 4	2.31 ± 0.27	51 ± 2	3.26 ± 0.15
SO + (SO)	Male	1751 ± 21 ${}^{\text{ab}}$	68.00 ± 0.60	41 ± 2	2.32 ± 0.13	53 ± 1	3.09 ± 0.10
SO + (PF)	Male	1754 ± 23 ${}^{\text{ab}}$	68.40 ± 0.77	42 ± 3	2.49 ± 0.20	61 ± 3	3.57 ± 0.18
SO + (T)	Male	1737 ± 21 ${}^{\text{ab}}$	68.45 ± 0.50	37 ± 4	2.13 ± 0.22	54 ± 1	2.98 ± 0.07
SO + (SO + T)	Male	1721 ± 19 ${}^{\text{ab}}$	67.95 ± 0.55	32 ± 3	1.88 ± 0.20	54 ± 2	3.14 ± 0.15
PF + (SO)	Male	1761 ± 23 ${}^{\text{ab}}$	68.25 ± 0.26	34 ± 4	1.92 ± 0.20	59 ± 2	3.33 ± 0.89
PF + (PF)	Male	1758 ± 10 ${}^{\text{ab}}$	67.82 ± 0.26	36 ± 2	2.02 ± 0.12	61 ± 3	3.46 ± 0.21
PF + (SO + T)	Male	1773 ± 11 ${}^{\text{a}}$	67.88 ± 0.78	39 ± 3	2.18 ± 0.17	55 ± 1	3.07 ± 0.04
T + (SO)	Male	1748 ± 35 ${}^{\text{ab}}$	67.71 ± 0.41	41 ± 3	2.36 ± 0.17	55 ± 1	3.29 ± 0.11
T + (T)	Male	1762 ± 5 ${}^{\text{ab}}$	66.52 ± 0.23 ${}^{\text{b}}$	40 ± 3	2.25 ± 0.16	60 ± 2	3.41 ± 0.10
T + (SO + T)	Male	1680 ± 21 ${}^{\text{bc}}$	67.80 ± 0.47	35 ± 3	2.09 ± 0.19	55 ± 2	3.23 ± 0.11
p values							
Fat type		0.013	0.201	0.237	0.425	0.001	0.002
Sex		0.000	0.914	0.398	0.001	0.004	0.001
Interaction, fat × sex		0.027	0.031	0.087	0.065	0.420	0.653

${}^{1}$ CW: carcass weight; CY: carcass yield; AFW: abdominal fat weight; AFY: abdominal fat yield; HLW: heart–liver weight; HLY: heart–liver yield. ${}^{2}$ SO: soy oil; PF: poultry fat; T: tallow. ${}^{\text{abcd}}$ The difference between the averages indicated by different letters in the same column are statistically significant ( p<0.050 ).

### 3.1 Field performance parameters

#### 3.1.1 Average live weight

It was seen that the broilers using PF and T instead of SO in their feeds showed an increasing tendency in ALW values (Table 6). This situation was more evident in the groups using T and SO for grower feeds, and some differences between various groups (ALW values of the PF + (PF), PF + (SO) and T + (SO + T) groups were higher than those of the SO + (SO + T) group) were found to be statistically significant ( P=0.043 ).

While the results of the present study were consistent with other findings of various researchers (Sell and Hodgson, 1962; Scaife et al., 1994; Azman et al., 2005) who reported an increase in ALW when T was used instead of SO, while they were not compatible with the results of other researchers (Dutra et al., 1991; Moura, 2003; Pinchasov and Nir, 1992) where ALW values did not change when animal fat was used instead of SO. This situation is thought to be caused by fixed energy : protein ratios as reported by various other researchers (Pinchasov and Nir, 1992; Quart et al., 1992; Leeson and Atteh, 1995; Pesti et al., 2002).

#### 3.1.2 Feed conversion ratio

It was observed that the FCR values of broilers improved when PF and T were used instead of SO in feeds (Table 6). This situation was more evident especially in the groups in which PF was used, and the differences between these groups (FCR values of PF + (PF) and PF + (SO) groups lower than SO + (SO + T) group) were found to be statistically significant ( P=0.034 ).

These results were supported by the data from the studies of Sell and Hodgson (1962) and Azman et al. (2005), who reported that FCR decreased when animal fat was used instead of SO. However, the results were not supported by the studies of Pinchasov and Nir (1992), Valencia et al. (1993) and Lara et al. (2003), who found that FCR was similar. This situation is thought to be caused by the use of SO in starter feeds and fixed energy : protein ratios as suggested by the researchers mentioned.

#### 3.1.3 Production efficiency factor

It was seen that the broilers using animal fat (PF and T) instead of SO in their feeds showed an increasing tendency in PEF values (Table 6). This situation was more evident especially in the groups in which only PF was used in feeds, and the difference between the groups (PEF values of PF + (PF) group higher than SO + (SO + T) group) was found to be significant ( P=0.011 ).

#### 3.1.4 Mortality

A decreasing tendency in SO groups and an increasing tendency in T groups were seen when the mortality values were evaluated (Table 6). This situation was more evident especially in groups where T and SO were used, but the differences between these groups were not found to be significant except for the difference in PF + (PF) and T + (SO + T) ( P=0.035 ).

The results were not consistent with the results of Pinchasov and Nir (1992) and Lara et al. (2003), who reported that the field performance did not change according to the fat source. This situation is thought to be caused by fixed energy : protein ratios as they suggested.

### 3.2 Slaughter performance parameters

#### 3.2.1 Carcass weight

CW values of male broilers were higher than those of female broilers ( P=0.000 ), and the differences were higher in the groups where SO was used in grower feeds. It was seen that the broilers fed with PF and T instead of SO in their feeds showed an increasing tendency in CW values (Table 7). This situation was more evident especially in the PF groups, but the differences between the groups were not found to be significant except for the PF + (SO + T) and T + (SO + T) groups ( P=0.013 ). Furthermore, the interaction between fat and sex was analysed, and the differences between various groups (CW of the T + (SO + T) group which were found to be lower than the PF + (SO + T) and T + (T) groups) were found to be significant ( P=0.027 ).

The results of the present study were not compatible with the results of the studies conducted by Andreotti et al. (2001) and Firman et al. (2008), who found no significant difference between CW of the SO and PF groups. This situation is thought to be caused by increased slaughter performance where using SO in starter feed and improved fat digestion with age increased as reported by Sell et al. (1986), Wiseman and Lesire (1987), and Aardsma et al. (2017).

#### 3.2.2 Carcass yield

A decreasing tendency was seen in CY of T, and an increasing tendency was seen in CY of SO. This situation is more evident especially between the SO and T groups for grower feeds, while the differences between these groups were not found to be significant ( P=0.201 ). Compared to feeding period and oil source used in feeds, CY values of the SO groups were higher than the other groups in grower feeds and an increasing tendency in the SO + PF and SO + T groups was observed for finisher feeds. However, the differences between the groups were not significant ( P=0.201 ).

CY values of male and female broilers were similar, and the effect of their sex on CY values was found to be insignificant ( P=0.914 ). An increasing tendency was seen in CY values of males except for the PF + (SO + T), SO + (SO + T), T + (T) and T + (SO + T) groups of males. However, the interaction between fat and sex was significant ( P=0.031 ) except for the PF + (SO + T) female and T + (T) male groups.

The results of the present study were not supported by the data of Andreotti et al. (2001) and Firman et al. (2008), who found no significant difference between CY of SO and PF groups. This situation is thought to be caused by increased slaughter performance and using SO in starter feed and improved fat digestion with increasing age.

#### 3.2.3 Abdominal fat weight

A decreasing tendency in AFW of the SO groups and an increasing tendency in AFW of PF and T groups were found in this study (Table 7). This situation was more evident for grower feeds, while the differences between the groups were not found to be statistically significant ( P=0.237 ).

AFW values of female and male groups were similar, and the differences between the groups were not significant ( P=0.398 ). In addition, the interaction between fat and sex was found to be insignificant ( P=0.087 ), while a decreasing tendency was seen in males except for the SO + (PF) group; bigger numerical differences were seen between various groups (females of the SO + (PF) group and females of the SO + (SO + T), SO + (T) groups with males of the SO + (PF) group).

The results of the present study were compatible with data of Andreotti et al. (2001) and Firman et al. (2008), while they were not compatible with the results of Sanz (1999), who stated that AFW was higher when animal fat was used. This situation is thought to be caused by fixed energy : protein ratio and non-high oil addition levels.

#### 3.2.4 Abdominal fat yield

A decreasing tendency in animal fat groups and an increasing tendency in SO groups were seen for AFY values (Table 7). This situation is more evident for grower feeds, while the differences between the groups were not found to be statistically significant ( P=0.425 ). In addition, numerical differences were seen between various animal fat groups, and the difference was bigger between the SO + (T) and SO + (SO + T) groups, none of which was found to be significant ( P=0.425 ).

AFY values of male broilers were lower than females ( P=0.001 ), and a decreasing tendency was seen in male broilers except for the PF + (SO) group. Similar to the fat treatment, the interaction between fat and sex was found to be insignificant ( P=0.065 ), while evident numerical differences were seen between various groups (females of the SO + (T) and SO + (PF) groups with males of the SO + (SO + T) and males of the PF + (SO) group).

The results of the present study were not supported by the data from the study of Sanz (1999), who stated that AFY was higher in groups in which animal fat was used. This situation is thought to be caused by fixed energy : protein ratio and non-high oil addition levels as similar to AFW.

#### 3.2.5 Heart–liver weight

An increasing tendency in the animal fat groups and a decreasing tendency in the SO groups in terms of HLW were seen (Table 7). This situation was more evident for grower feeds as the differences between various groups were statistically significant ( P<0.05 ), and HLW values of the T + (T) group were higher than the SO + (SO) and SO + (T) groups ( P=0.001 ).

HLWs of male broilers were higher than females ( P=0.004 ) and HLW values increased in males except for the SO + (SO + T) and PF + (SO + T) groups. However, a significant interaction was not found between fat and sex ( P=0.420 ), while numerical differences were seen in various groups (males of the SO + (PF), PF + (PF), PF + (SO) and T + (T) groups with the females of the SO + (SO), SO + (T) and T + (SO + T) groups).

#### 3.2.6 Heart–liver yield

A decreasing tendency in the SO groups and an increasing tendency in the animal fat groups were seen for HLY (Table 7). This situation was more evident for grower feeds, and the differences between various groups (the SO + (PF), T + (T) and SO + (T) groups) were found to be significant ( P=0.002 ).

HLY values were lower in male broilers than females ( P=0.001 ), and HLY decreased in males except for the SO + (SO) and SO + (PF) groups. However, the interaction between fat and sex was not found to be significant ( P=0.653 ), while bigger numerical differences were seen between various groups (females of the SO + (T), PF + (SO) and T + (T) groups with males of the SO + (SO) and PF + (SO + T) groups).

## 4 Conclusions

According to the results of this study, an improvement in field performance parameters except for mortality were observed when animal fat (PF and T) was used instead of SO in feeds with the fixed energy : protein ratio. This situation was more evident in grower feeds. A similar situation was not observed in the slaughter performance parameters except for CW, HLW and HLY. However, sex was found to affect the slaughter parameters except for CY and AFW; higher CW and HLW and lower AFY and HLY were observed due to higher CW in males. The interactions between the type of the fat and sex were not found to be significant except for CW and CY. In conclusion, it can be said that PF and T alone or their combinations with SO could be used to meet metabolic energy needs, provide more economical production, and increase the usage of by-products such as PF and T when vegetable oil prices are not considered appropriate. It is thought that animal fats of ruminant origin that produced BSE-like disease-free processes can be used in feeding of different species, as stated in legal regulations. However, further study is necessary.

## Data Availability

All necessary data are provided within the article.
